# Supplementary material for: Hydroxycinnamic Acid Derivatives from Coffee Extracts Prevent Amyloid Transformation of Alpha-Synuclein
Source: Biomedicines. 2022 Sep 12;10(9):2255. doi: 10.3390/biomedicines10092255 (PMC9496549; doi:10.3390/biomedicines10092255)
Supplement: Supplementary file 1 [file biomedicines-10-02255-s001.zip › biomedicines-1857936-supplementary.pdf]

Supplement

Hydroxycinnamic Acid Derivatives from Coffee Extracts Prevent Amyloid Transformation of Alpha-Synuclein

M.V. Medvedeva[1], N.A. Kitselovskaya[2,3], Y.Y. Stroylova[4], I.A. Sevostyanova[4], A.A. Saboury[5] and V.I. Muronetz[1,4*]

1 Faculty of Bioengineering and Bioinformatics, Lomonosov Moscow State University, 119234 Moscow, Russia

2 Department of Systems Biology, National Medical Research Center for Obstetrics, Gynecology and Perinatology of Ministry of Healthcare of Russian Federation, 117997, Moscow, Russia

3 Federal State Budgetary Institution "Federal Research And Clinical Center Of Physical-Chemical Medicine", Federal Medical Biological Agency, 119435, Moscow, Russia

4 Belozersky Institute of Physico Chemical Biology, Lomonosov Moscow State University, 119234 Moscow, Russia

5 Institute of Biochemistry and Biophysics, University of Tehran, Tehran 1417614335, Iran

* Author to whom correspondence should be addressed, e-mail: vimuronets@belozersky.msu.ru

Supplemental Figures

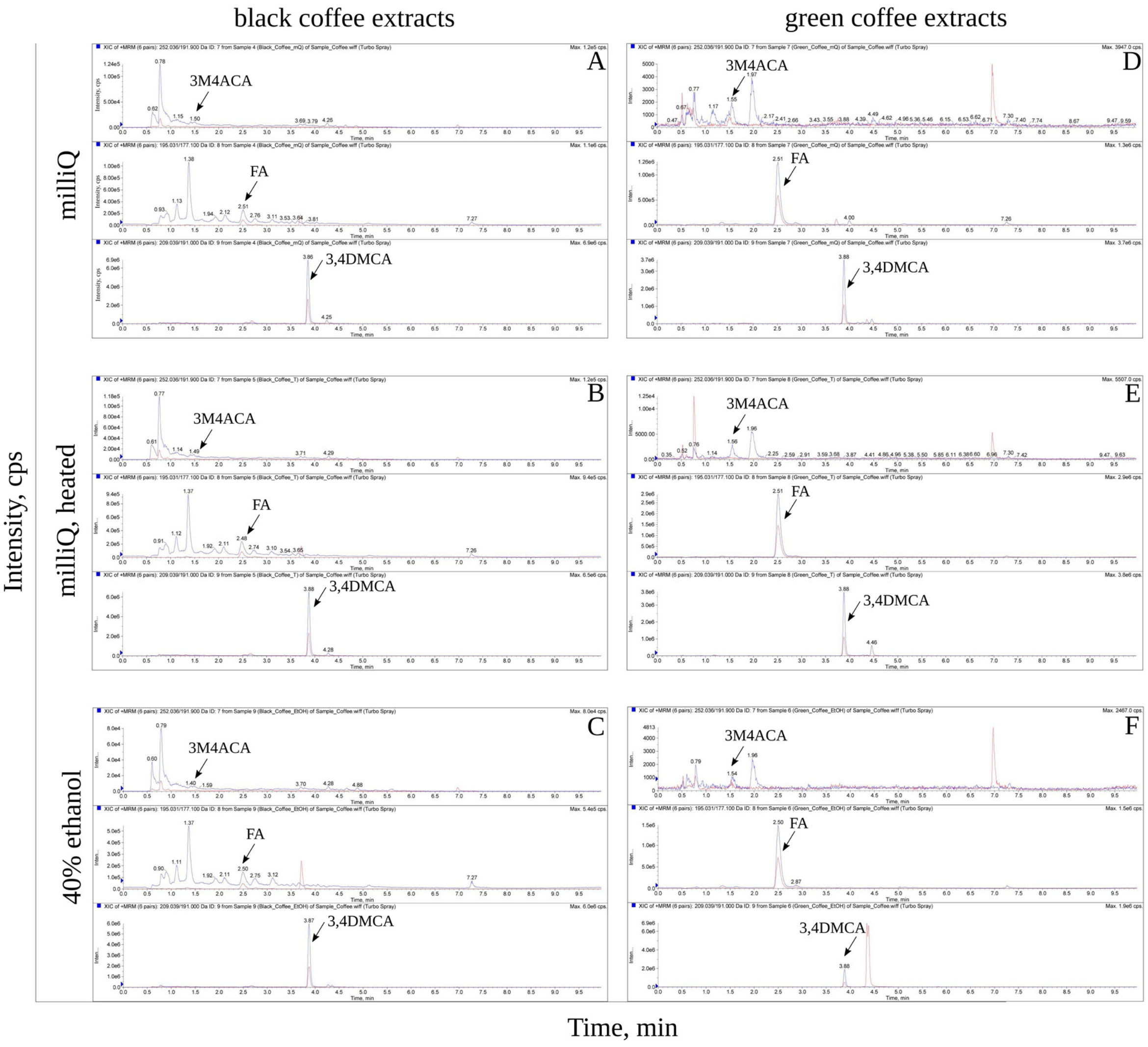

Figure S1. Chromatograms of coffee extracts: aqueous extract of black coffee (**A**); aqueous extract of black coffee obtained by boiling (**B**); ethanol extract of black coffee (**C**); aqueous extract of green coffee (**D**); aqueous extract of green coffee obtained by boiling (**E**) and ethanol extract of green coffee (**F**).

Supplemental Tables

Table S1

Chromatographic gradient.

Time, min	Solvent A, %	Solvent B, %	Flow rate, µl/min
0.00	85	15	400
5.00	5	95	400
7.00	5	95	400
7.01	85	15	400
10.0	85	15	400

Table S2

Concentrations of test substances in calibration standards.

Calibration dependence level	Ferulic acid concentration, ng/ml	3,4-dimethoxycinnamic acid concentration, µg/ml	3,4-dimethoxycinnamic acid concentration, µg/ml
Level 1	1.25	0.05	0.05
Level 2	2.50	0.10	0.10
Level 3	5.00	0.50	0.50
Level 4	10.00	1.00	1.00
